# A chronicle review of new techniques that facilitate the understanding and development of optimal individualized therapeutic strategies for chordoma

**DOI:** 10.3389/fonc.2022.1029670

**Published:** 2022-11-16

**Authors:** Chenglong Zhao, Tao Tan, E. Zhang, Ting Wang, Haiyi Gong, Qi Jia, Tielong Liu, Xinghai Yang, Jian Zhao, Zhipeng Wu, Haifeng Wei, Jianru Xiao, Cheng Yang

**Affiliations:** ^1^ Spinal Tumor Center, Department of Orthopedic Oncology, Changzheng Hospital, Shanghai, China; ^2^ Department of Orthopedics, 905 Hospital of People’s Liberation Army Navy, Shanghai, China

**Keywords:** chordoblast, organoid, NGS—next-generation sequencing, precision treatment, drug discovery

## Abstract

Chordoma is a rare malignant bone tumor that mainly occurs in the sacrum and the clivus/skull base. Surgical resection is the treatment of choice for chordoma, but the local recurrence rate is high with unsatisfactory prognosis. Compared with other common tumors, there is not much research and individualized treatment for chordoma, partly due to the rarity of the disease and the lack of appropriate disease models, which delay the discovery of therapeutic strategies. Recent advances in modern techniques have enabled gaining a better understanding of a number of rare diseases, including chordoma. Since the beginning of the 21st century, various chordoma cell lines and animal models have been reported, which have partially revealed the intrinsic mechanisms of tumor initiation and progression with the use of next-generation sequencing (NGS) techniques. In this study, we performed a systematic overview of the chordoma models and related sequencing studies in a chronological manner, from the first patient-derived chordoma cell line (U-CH1) to diverse preclinical models such as the patient-derived organoid-based xenograft (PDX) and patient-derived organoid (PDO) models. The use of modern sequencing techniques has discovered mutations and expression signatures that are considered potential treatment targets, such as the expression of Brachyury and overactivated receptor tyrosine kinases (RTKs). Moreover, computational and bioinformatics techniques have made drug repositioning/repurposing and individualized high-throughput drug screening available. These advantages facilitate the research and development of comprehensive and personalized treatment strategies for indicated patients and will dramatically improve their prognoses in the near feature.

## Introduction

Chordomas are extremely rare malignant bone tumors originating from the remnant notochord (1, 2). The estimated annual incidence is approximately 0.8–1 out of 1,000,000, with male dominance (3, 4). Almost all primary tumors accumulate in the axial skeleton, with a predilection of the sacrum and the clivus/skull base. According to the WHO classification (5th edition), chordomas are pathologically classified into three subtypes: conventional (including the chondroid), poorly differentiated, and dedifferentiated (5). The conventional subtype is the most common, accounting for more than 95% of all cases (6).

Chordomas are considered as low- to intermediate-grade malignancies with a moderate growth rate, but with locally aggressive features. The ideal treatment is en bloc resection of the primary tumor without metastasis (7, 8). However, most patients with chordoma lack the typical clinical symptoms in the early stage due to the indolent characteristic of this tumor. Most chordoma tumors are large at the first diagnosis, with a pseudo-capsule that is vulnerably violated intraoperatively. On the other hand, as most chordoma tumors originate from the cranio-caudal ends of axial skeletons, vital structures such as the pituitary, spinal cord or cervical/sacral nerve roots, vertebral/iliac arteries, rectum, and the sigmoid colon are often involved or even enrolled by the tumor mass, which constitutes a great challenge in the surgical intervention of this malignancy. Subtotal resection with adjuvant radiotherapy is considered as an alternative treatment, but insufficient resection of the tumor often leads to a high risk of subsequent local recurrence or even metastasis (9).

The 5-year recurrence-free survival (RFS) of chordoma is over 50% for patients with adequate resection margins, but the rate may drop dramatically to less than 20% in the presence of contamination (7, 9, 10). Few advanced cases can be cured radically by surgery, and systemic treatment is required, but the effect is limited (6, 8, 9). Conventional chordomas are considered as indolent to cytotoxic chemotherapy, while the poorly differentiated and dedifferentiated subtypes are considered as sensitive to chemotherapy. Systemic treatment is considered as upfront management for chordoma by most researchers and clinicians, but more evidence-based verification is required (11–14).

To some extent, the current clinical dilemma is attributed to the lack of basic research on chordoma compared with other tumors. Given the low occurrence rate and the indolent biological feature of chordoma, it is usually difficult to develop stable cell lines and suitable xenograft animal models, which hinders gaining a deeper understanding of the intrinsic mechanism of the biological processes of chordoma. It was not until 2001 that the first chordoma cell line was established and well characterized (15). With the joint efforts of clinicians, biologists, pharmacologists, and numerous other multidisciplinary teams, chordoma research has made great progress in the new century. New treatment strategies, including tumor vaccines, targeted therapies, and immunotherapies, have been developed and have shown promising prospects. In the current review, we focus on the progress in the basic research of chordoma in recent years and its far-reaching impact on clinical practices. In addition, we provide an overview of modern individualized drug screening systems for chordoma.

## The research tools: Chordoma cell lines and animal models

The importance of cell lines and animal models for disease research cannot be doubted. However, since the identification of the first case in 2000, chordoma research has mainly focused on the clinical symptoms, radiological expressions, and pathological manifestations. Compared with other types of malignant tumors, the lack of understanding of the pathogenesis of chordoma and effective medical therapies is the partial reason for the delayed establishment of model systems. There is limited basic research in identifying karyotype abnormalities and characteristic molecules from clinical samples. It was not until 2001 that the first human chordoma cell line, U-CH1, was established (15), and about 10 years later, the first xenograft model was reported (16). Today, over 20 chordoma cell lines and several different types of animal models have been established, which substantially expands our understanding of the pathogenesis of chordoma.

### Cell lines

The U-CH1 cell line was developed from a sacrum tumor of a 46-year-old male patient who received radiotherapy for local recurrence of the tumor 4 years after the primary resection. Further characterization proved typical physaliphorous morphological features and stable expression of several markers, including Brachyury, vimentin, and cytokeratin (15). Since its establishment, the U-CH1 cell line has become one of the most widely used chordoma cell lines worldwide. Several interesting intrapersonal models of cell line families with unique characteristics have been established. The U-CH11 and U-CH11R cell lines were established from the primary and the recurrent sacral chordoma (recurred 4 years after primary resection) of the same patient, respectively (17). The primary tumor tissue, the U-CH11 cell line, and the corresponding recurrent cell lines were compared using RNA sequencing (RNA-seq). The results demonstrated that transcriptomic reprogramming occurred during chordoma recurrence, which did not derive from genomic events. Similar conclusions were also drawn from another series of cell lines originating from the same patient. U-CH17P, U-CH17M, and U-CH17S were established from the primary site, lung metastasis, and skin metastasis, respectively (18). Although there was some loss of genetic variations compared with the parental chordoma tissues, such cell line model systems are meaningful for the investigation and understanding of the intrinsic mechanisms underlying the process of tumor progression.

Compared with cell lines established from sacrococcygeal tumors, clivus-originated chordoma cell lines are even more difficult to breed. Lucia et al. reported a technical note on establishing three clival chordoma cell lines from patients, but the cells were not permanent (19): the cells underwent crisis with continued passaging for about 40 generations. Owen et al. established the first permanent human clival chordoma cell line, UM-Chor1, and successfully transduced it with a luciferase lentiviral vector (20). A para-sacral xenograft model in non-obese diabetic/severe combined immunodeficiency (NOD/SCID) mice was also built, which showed slow growth *via* bioluminescence. The lack of clival chordoma cell lines could possibly be explained by the role of aggressive telomerase in chordoma and the destruction or damage of tumor cells intraoperatively (19). Gellner et al. thoroughly analyzed such suspects and successfully bred the clival cell line MUG-CC1 using a full endoscopic technique under suitable culture conditions (21). Since non-tumorigenic notochordal cell lines are unlikely to be available in near future, their team also creatively presented a non-tumorigenic, spontaneously established lymphoblastoid cell line originating from the same chordoma patient (termed as MUG-CC1-LCL) for comparative analysis. Recently, Kino et al. have established a novel skull base chordoma cell line, TSK-CHO1 (22), which has been proven to be neoplastic and which exhibited pleomorphic features. It was also revealed that the TSK-CHO1 cell line secretes Brachyury and SOX9 into conditioned medium (CM), which can further induce human dental pulp stem cell differentiation and promote the production of hyaluronic acid and type II collagen. This new cell line is expected to be used for elucidating the pathogenesis of skull base chordoma and investigating the mechanism underlying the production of fibrocartilage.

Most currently available cell lines have been derived from conventional chordomas, but the origin of the other subtypes is unknown. Kim et al. successfully established a chordoma cell line from a patient with recurrent dedifferentiated chordoma and named it DTC (23). Compared with U-CH1 cells, DTC cells showed a unique polygonal morphology and higher clonogenic activity. Compared with conventional chordomas, a distinct expression spectrum was also identified, with a high expression of platelet-derived growth factor receptor-β (PDGFR-β) and stemness- and epithelial-to-mesenchymal transition (EMT)-related proteins, but low levels of Brachyury and cytokeratins. DTC cells were also demonstrated to have a high surface expression of CXCR4 and the capacity to form xenograft subcutaneous tumors in nude mice.

### 3D cultures of chordoma cell lines

It is well documented that chordoma cells are embedded in a massive extracellular myxoid matrix *in vivo*. The extracellular components not only function as a passive scaffold within the tumor architecture but also play a more active role, thus facilitating the communication between cells and participating in the regulation of cellular proliferation, differentiation, and motility. Therefore, 3D cultures are considered to have obvious superiority over traditional 2D cultures (24, 25). Cao et al. reported their experience using 3D cultures of chordoma cell lines (26). They cultured three cell lines (U-CH1, CH8, and GB60) the growth factor-reduced Matrigel by using an assay medium with the addition of extra growth factor 12 (DMEM/F12) containing 2% horse serum, 0.5 g/ml hydrocortisone, 100 ng/ml cholera toxin, 10 g/ml insulin, 100 U/ml penicillin G, and 100 mg/ml streptomycin after the tumor was seeded on the reconstituted basement membrane. The clusters and acini-like spheroids formed by the cells partially restored the *in vivo* morphology. Locquet et al. prepared 3D cellular models using cell lines representative of three different stages of the disease: U-CH12 for primary, U-CH1 for relapsed, and CH22 for metastatic tumor (27). Their tumor spheroids recapitulated the main histological and morphological features, as well as the radioresistant environment of chordoma.

The 3D cultures of the patient-derived primary tumor cells were named as organoids (patient-derived organoids, PDOs). More advantages of organoids over conventional 2D patient-derived cell cultures (PDCs) have been recognized and documented (28–30). However, similar obstacles, such as indolent tumor growth and difficulty in stable passage, need to be overcome by establishing chordoma organoid models in tumor cell lines. Scognamiglio et al. reported their experience in predicting the response of patients to the checkpoint inhibitors (ICIs) programmed cell death 1/programmed death ligand 1 (PD-1/PD-L1) (31). In their brief communication, they reported the first evidence of using chordoma PDOs to examine individual responses to treatment. In a recent report, Shihabi et al. have documented their experience in establishing the PDO model with a 100% success rate, stating that the model mimicked the immunohistopathological characteristics of the parental tumor (32). Furthermore, they built an effective and timely high-throughput drug screening platform with their PDO model. Additionally, the PDO model was obviously more cost-effective than the patient-derived xenograft (PDX) model. Details are presented in the subsequent section.

Collectively, the 3D culture and PDO models can serve as important supplements to traditional *in vitro* and *in vivo* animal models, which can greatly accelerate the study of such a rare disease and help in the discovery of potential therapeutic drugs. Details are presented in subsequent discussions.

### Animal models

Two types of animal models have been established: the xenograft tumor model and the zebrafish tumor model. The former was derived based on chordoma cell lines or patient-derived tumors, while the latter is mostly a spontaneous animal model used to facilitate the understanding of the mechanisms of tumor initiation and progression.

Wesley et al. injected JHC7 cells into immunodeficient mice subcutaneously and successfully constructed the first cell line-derived (CLD) chordoma xenograft animal model (16). Further experiments demonstrated that the injected tumor vividly resembled the parental tumor phenotype. Siu et al. established the first serial transplantable PDX model (33). Compared with the CLD model, the PDX model more closely mimicked the original tumor and was more valuable for effective therapeutics (34). Using this model, Siu et al. (35) further evaluated the efficacy of the epidermal growth factor receptor (EGFR) inhibitor erlotinib *in vivo*. Subsequently, several PDX models reflecting different tumor characteristics were established, including primary or recurrent tumors involving the cervical spine or sacrum (36–38). However, all these tumors were subcutaneously injected; *in situ* transplanted models continue to be pursued.

Diaz et al. (34) established a clival chordoma xenograft to mimic *in situ* tumor characteristics (34). The tumors were harvested intraoperatively, digested, resuspended, and mixed with Matrigel. NOD/SCID gamma (NSG) mice were prepared by scraping the outer cortex of the parietal bone with a scalpel. The obtained tumor cells were implanted into the subcutaneous epicranial space above the posterior parietal bone and the sub-occipital musculature thereafter. Although not on each PDX model, bony invasion was observed in each generation, which partially restored the clinical features of patients compared with the primary tumor. Salle et al. (39) developed an orthotopic primary PDX model with tumors implanted in the lumbosacral area. Compared with the clivus, the lumbosacral area was preferred in order to allow tumor growth and avoid animal suffering and mortality related to the growth of the tumor. The success rate of tumor engraftment was significantly increased using this orthotopic technique, from about 30%–40% to 60%–80%.

Compared with the xenograft model, the tumorigenic model was more suitable for the investigation and understanding of tumor development and progression. In the new century, zebrafish has emerged as a powerful genetic model of human cancer with the advantages of high genetic conservation, operable genetic manipulations, and feasibility for observation (40). Burger et al. reported the first zebrafish model for chordoma research (41). They built a notochord-specific expression of HRASV12 in the Gal4/upstream activating sequence (UAS) system, which was one of the first inducible transgenic methods providing a unique opportunity to induce oncogene expression in a tissue-specific manner in zebrafish. Although no evidence of *HRAS* mutations has been reported in human chordomas, their model firstly observed a chordoma-like tissue malformation of the notochord with positive expressions of Brachyury and cytokeratin. A primary distinction observed between the examined zebrafish notochord tumors and the human chordoma is the rapid phenotype onset in the fish model compared with the slow growth of human cancer. These features facilitated an application in high-throughput drug screening. With this *in vivo* platform, the authors further investigated other potential drivers of chordoma initiation (42). Notably, their data imply that Brachyury per se might be insufficient to initiate chordoma and that the active receptor tyrosine kinase may potently induce a chordoma phenotype.

Several other genes were examined for their ability to induce tumor initiation in zebrafish models. The upregulation of the metastasis-associated gene *PRL-3* and the downregulation of the transforming growth factor-β family member *TGFB3* have been proven to be correlated with chordoma formation (43). Although there was no direct evidence of the contribution of *FAS*/*FASL* dysregulation to chordoma formation, the knockdown of this pair of genes in zebrafish strikingly impaired notochord formation in the zebrafish model, suggesting their possible involvement in chordoma occurrence (44). In addition to these genetic manipulated models, an interesting finding should be noted. Cooper et al. (45) reported a series of 24 cases of spontaneous primary intestinal chordomas in zebrafish and nine cases of spontaneous vertebral chordoma. The former represents a novel tumor type that had not been previously described in any species.

At present, most chordoma cell lines and several serial passable PDX models are well documented by the Chordoma Foundation (https://www.chordomafoundation.org/research/disease-models/) and are available to researchers. With the help of these valuable research models, chordoma research has made great strides and accelerated the development of comprehensive therapeutic drug discovery.

## Leap to the next generation: Advances in genetic research

The identification of driver mutations and downstream regulatory abnormalities in malignancies is beneficial to revealing the intrinsic mechanisms of tumor initiation, progression, and recurrence and has dramatically changed the treatment landscape for many tumors. Multiple techniques for genetic analysis have been applied to karyotype analysis, comparative genetic hybridization (CGH), high-throughput microarray, and the NGS of chordoma. Some recent studies have applied modern sequencing techniques to chordoma investigation, including single-cell RNA sequencing (scRNA-seq), whole-genome bisulfite sequencing (WGBS), transposable accessible chromatin by high-throughput sequencing (ATAC-seq), and Hi-C.

### Early exploration of cytogenetic changes in chordoma

Due to restrictions in the investigation techniques, only limited studies with 18 chordoma cases focusing on cytogenetic changes were reported before 2000 (46). Few specific or characteristic chromosomal anomalies have been determined, except in one study by Butler et al., which reported the results of cytogenetic analysis of five chordomas (46). Only random abnormalities in one tumor cell out of 100 cells from Patient No. 5 were identified. However, their study identified elongation, but not reduction as in other tumors, of telomere length and an increase of telomerase activity. In the new century, several studies have demonstrated loss of heterozygosity for different loci in chordoma. In Klingler’s cohort of 12 patients with chordoma, microsatellite instability (MIN) was demonstrated in six patients and loss of heterozygosity (LOH) for at least one locus in two patients (47). Several other LOH and comparative genomic hybridization (CGH) studies demonstrated more chromosome abnormalities that showed a feature of more loss than gain, including loss of 1p36, 7q33, and 9p21 as an important mechanism for tumor development (48–50). Furthermore, candidate genes mainly located in the locus of interest were mapped and identified using PCR, gene chips, and immunohistochemistry (IHC). At the same time, researchers firstly attempted to use imatinib mesylate, a selective tyrosine kinase inhibitor (TKI) of *c-KIT* and platelet-derived growth factor receptors, for patients with chordoma as it was shown to be highly effective in gastrointestinal stromal tumors (GIST) (51). Although some benefits in tumor control have been demonstrated in clinical practice, most of the samples were negative with activating mutations but overexpression of the receptor tyrosine kinase (RTK) genes, which can be identified with RT-PCR or IHC (52–54). Since then, many studies concerning the expression and functional analyses of RTK and other candidate genes have been performed with tissue microarray, with the results indicating a promising pattern for corresponding inhibitors in the treatment of chordoma (55–59).

High-throughput microarray has further promoted our understanding of such a rare tumor. The first array-based study characterizing DNA copy number changes in chordoma identified copy number alterations in all samples (50). Deletions were more common than gains, and no high-level amplification was found. Henderson et al. firstly drew a molecular map of mesenchymal tumors and identified a gene expression signature of chordoma through gene expression microarray (GEM) in a machine learning model (60). Among which, the T-box transcription factor (*TBXT*) Brachyury was first identified, which was mainly expressed in the developing notochord and strongly linked the embryonic structure with chordoma. Pillay et al. identified *TBXT* amplification in both familial and sporadic chordomas, finding that it was the rs2305089 polymorphism that altered the binding ability of *TBXT*, which is known to be closely correlated with tumor initiation in individuals of European ancestry (61). Further mutation studies also identified other common variants of *TBXT* with risk of chordomas, such as rs3816300 in sporadic chordomas and rs1056048 in familial cases (62). Since then, large numbers of studies have verified the important roles of Brachyury in the initiation, progression, and prognosis of chordoma. As the results have been well collective and compared in several other reviews (63–65), we will not discuss them herein. However, a recent randomized, double-blind, placebo-controlled phase II trial failed to observe advantages of the yeast–Brachyury vaccine combined with standard-of-care radiotherapy in locally advanced unresectable chordoma (66). Although the results of the yeast–Brachyury vaccine are not consistent, the idea of targeting Brachyury with other immune or targeted methods for chordoma therapy is still worth further exploration.

Since only a few driver mutations but dramatic functional gene expression signatures were identified in chordomas, exploration of the epigenetic changes was conducted (67, 68). Rinner et al. (69) reviewed 10 chordoma samples and found that the PI3K pathway played a potential important role in the development of chordoma. They first provided evidence of the DNA methylation of tumor suppressor genes in chordoma, suggesting that they could serve as markers for its early detection. Another study (70) identified a subset of probes that were differentially methylated between recurrent and non-recurrent chordomas. Identification of the DNA methylation features of chordoma has provided a wealth of information in establishing useful biomarkers for diagnosis, prognosis prediction, and disease monitoring, and these biomarkers could also be potential therapeutic targets for this rare tumor.

### NGS techniques provide a deeper understanding of chordoma

Due to the urgent need to gain more thorough insights into the molecular biology and genetics of chordoma, ultra-deep NGS analysis was performed (as listed in **Table 1**). Fischer et al. (71) performed the first panel-based NGS study in nine patients with chordoma for the mutations of 48 cancer genes, but failed to identify somatic mutations in “hotspots” of genes known to be involved in cancer development; very low mutation rates of *KDR*, *KIT*, and *TP53* were determined. Another panel-based study of 341 key cancer-associated genes was performed in 23 chordoma samples (72). The results revealed non-random copy number losses across the genome and a very low mutation rate, with an average of 0.5 mutation per sample. However, about 40% of the mutation events were grouped as chromatin regulatory genes, including *SETD2*, *PBRM1*, *ARID1B*, and *SMARCB1*, and the co-occurrence of alterations in *CDKN2A*/*CDKN2B* was fairly common. These findings hinted that chordoma might belong to the C class tumors in terms of copy number changes whose oncogenic signature was a non-random multiple copy number loss across the genome, and these genomic aberrations frequently alter chromatin regulatory genes.

**Table 1:** Next-generation sequencing (NGS) studies on chordoma.

Reference	No. of patients	Sequencing technique	Tumor sites	Primary/recurrent	Subtypes
Pillary et al. (59)	20 (European ancestry)	WES	NA	NA	NA
Fischer et al. (71)	9 (Caucasians)	NGS (the TruSeq Amplicon Cancer Panel)	3 skull base, 4 spine, and 2 sacrum/coccyx	Primary	Conventional (7 classic and 2 chondroid)
Wang et al. (72)	24	NGS (MSK-IMPACT)	3 spine, 18 sacrum, and 3 pelvic	21 primary and 3 recurrent	Conventional
Bell et al. (73)	14	RNA-seq analysis	Skull base	NA	13 conventional (including 3 chondroid) and 1 dedifferentiated
Sa et al. (74)	10	WES in 8 patients and RNA-seq analysis in 5 patients	Skull base	6 primary and 4 recurrent	Conventional (5 classic and 5 chondroid)
Bell et al. (75)	12	RNA-seq analysis in 12 patients	Spine	Primary	Conventional
Tarpey et al. (76)	104	WGS in 11 patients, WES in 26 patients, and NGS (targeted sequencing) in 67 patients	19 skull base, 25 spine, 50 sacrum/coccyx, 3 extra axial, and 7 unknown	NA	89 conventional, 5 dedifferentiated, 4 poorly differentiated, and 6 unknowns
Gröschel et al. (77)	11	WES in 9 patients and WGS in 2 patients	3 skull base, 5 spine, and 3 sacurm	NA	NA
Hung et al. (12)	4	WES in 4 patients	NA	4 primary and 1 Paired metastatic	Dedifferentiated
Zhu et al. (78)	11	NGS (MSK-IMPACT)	NA	NA	NA
Bai et al. (79)	80 (Chinese patients)	WGS	Skull base	80 primary and 11 paired recurrences	78 conventional (including 14 chondroid) and 2 dedifferentiated
Mattox et al. (80)	32	WES	14 spine, 15 sacrum, 2 pelvic, and 1 chest	31 primary and 1 metastatic	NA
Yepes et al. (81)	138 (European ancestry)	WES	55.7% skull base, 23% spinal, and 20.3% sacral	NA	NA
Wen et al. (82)	3	NGS (MSK-IMPACT)	Extra-axial	Primary	1 conventional and 2 poorly differentiated
Xu et al. (83)	8 (Chinese patients)	WES	Sacrum	4 primary and 4 recurrent	NA
Duan et al. (84)	6 (Chinese patients)	scRNA-seq	3 spine, 1 sacrum, and 2 skull base	NA	NA

WES, whole-exome sequencing; NGS, next-generation sequencing; RNA-seq, RNA sequencing; WGS, whole-genome sequencing; scRNA-seq, single-cell RNA sequencing; NA, not available.

Restricted by the limited number of genes investigated in panel-based studies, a number of studies used whole-exome and whole-genome sequencing (WES and WGS, respectively), as these techniques can shed further light into the pathogenesis of and potential treatment options for chordoma. Jason et al. (74) combined WES and whole-transcriptome sequencing on 10 clivus chordomas and identified a novel gene fusion of *SMAD5*–*SASH1* for the first time in the skull base, which can be employed as a therapeutic and prognostic marker (74). Tarpey et al. (76) constructed a driver landscape of 104 cases of sporadic chordoma with 11 WGS, 26 WES, and 67 panel-based sequencing. WGS/WES studies were used as the discovery cohort and the remaining studies used as the validation cohort. This study identified somatic duplications of *TBXT*, recurrent mutations of *PI3K* signaling genes, and driver events in chromatin modeling genes, consistent with the results of the aforementioned studies. In addition, they also identified non-random enrichment of truncating mutations in the lysosomal trafficking regulator (LYST) protein for the first time and found that the expression of LYST was correlated with the function of lysosomes, a histological hallmark of physaliphorous vacuole-packed cells that characterize chordoma. *LYST* may work as a cancer gene in chordoma and prove to be an adjunct diagnostic marker.

The genomic features differed between ethnicities, tumor locations, and pathological subtypes. As aforementioned, the rs2305089 polymorphism in *TBXT* was associated with a sixfold increase in the risk of developing chordoma in a European population, but it was not found to be correlated with an increased risk in East Asians (81). On the other hand, somatic duplications of *TBXT* and mutations in *PI3K* signaling genes are rare in skull base chordomas, which was different from that observed in sacral chordoma. In a Chinese patient-based study of sacral chordomas, Xu et al. (79) identified a new *CLDN9* T120A substitution as a potential oncogene in indicated populations, which had not been previously reported. Previous analyses have identified pathological changes in chordomas (12). Compared with benign notochordal cell tumors, the conventional and dedifferentiated chordoma subtypes showed an increased genomic instability (78). In dedifferentiated chordomas, Brachyury was expressed in the conventional/chondroid components, but was completely lost in the dedifferentiated component. Poorly differentiated chordomas are characterized by the loss of *INI1*/*SMARCB1* and may also represent a discrete entity with a more aggressive phenotype, which is more similar to rhabdoid tumors. However, driver gene alterations are critical in tumor initiation and progression, but may not be involved in tumor dedifferentiation and high-grade transformation (82).

WES/WGS in chordoma tissues demonstrate a low-frequency mutation rate compared with that in other cancer tissues. Apart from genetic alterations, epigenetic regulators and chromatin spatial organization are also crucial for gene regulation by regulating the accessibility of DNA to sequence-specific binding proteins and bringing distant promoters, enhancers, and other *cis*-regulatory regions together (85, 86). Meng et al. (87) performed a multi-omics analysis with RNA-seq, ATAC-seq, and Hi-C and found that the bone microenvironment played an important role in chordoma tumorigenesis. In addition, they identified carbonic anhydrase II (*CA2*) as a novel therapeutic target in chordoma. ScRNA-seq could elucidate the mechanisms underlying carcinogenesis and the molecular features of cancers. Duan et al. (84) first delineated the transcriptomic landscape of chordoma using scRNA-seq. Their results identified six subclusters of chordoma cells, which exhibited properties of an epithelial-like extracellular matrix, and stem cells with immunosuppression. They also identified a strong immunosuppressive effect exerted by regulatory T cells (Tregs) and M2 macrophages and an enhanced TGF-β signaling pathway in tumor progression. Immune therapy is considered as a promising strategy for tumor control, and several clinical cases have been reported to respond to immune ICIs. The study of Duan et al. partially explained the rationale for immune therapy in patients with chordoma, although in-depth research is still required.

With the development of NGS techniques, the design of individualized therapy using comprehensive genomic analysis has been attempted and clinically practiced. Screening for hotspot mutations in cancer-associated genes prior to a personalized treatment approach in patients with limited therapeutic options can provide a rationale for genomics-guided therapy. Liang et al. (88) reported their experience on the combined use of WES and RNA-seq in four patients with chordoma and found that the incorporation of different sequencing techniques could help further clarify which DNA alterations are expressed or could be used as therapeutic targets. Another more detailed analysis of WES/WGS-guided therapy was reported by Stefan et al. (77). The authors observed that advanced chordomas were characterized with frequently impaired DNA repair *via* homologous recombination (HR) and with mutations of HR-related genes, including *BRCA2*, *NBN*, and *CHEK2*. Treatment with the poly(ADP-ribose) polymerase (PARP) inhibitor showed promising clinical prospects for such patients. However, one case gained olaparib resistance after a 10-month treatment, when tumor reprogression occurred due to a second mutation of the p.T910A allele and restored the activity of PARP. Gene expression-based estimates of immune cell abundance have the potential to identify patients that may respond to ICI treatment (89–91). Growing evidence has also shown responses to ICIs in tumors deficient in SWI/SNF chromatin remodeling genes (92, 93). Therefore, poorly differentiated chordoma with a characteristic *SMARCB1* deficit is a potential candidate for ICI treatment. Williamson et al. (94) performed multi-omics sequencing including WGS, RNA-seq, and WGBS and successfully identified a recurrent pediatric poorly differentiated chordoma with features of single copy losses affecting *SMARCB1*, hypomethylation of the *TBXT* promoter, overexpression of the Brachyury tumor antigen and *CD274* (PD-L1), and CD8^+^ T-cell, CD79a^+^ B-cell, and plasma cell infiltration. All these features hinted of a potential effect of ICIs, which have been used in clinical practice and with proven satisfactory outcomes.

Collectively, genetic research on chordoma using multiple techniques revealed a relatively quiet cancer genome driven by a limited repertoire of cancer genes, and epigenetic regulations may play an important role in tumor initiation and progression. The combination of multi-omics analyses helps in the comprehensive understanding of the tumor landscape and facilitates the design of individualized treatment strategies.

## New era: Modern personalized precision drug treatment

At present, surgery remains the mainstay of treatment for chordoma. However, complete tumor resection is not always available; therefore, there is an urgent need to develop new therapeutic options for this tumor (1, 8, 9). Conventionally, chordoma is considered resistant to chemotherapy, and only anecdotal cases have provided limited evidence for the clinical employment of cytotoxic agents (6, 95). Recent studies have reported responses to agents such as anthracyclines, alkylating agents, cisplatin, and etoposide in the ultra-rare subtype of dedifferentiated chordoma (96–98). The combined use of cytotoxic drugs as sensitizers for radiotherapy or targeted therapy has also garnered attention (99, 100). Through sequencing efforts in chordoma, targeted therapies have shown promising prospects in tumor management. Nevertheless, unclear indications for individualized application of the limited available drugs have hindered comprehensive treatment of patients with chordoma. Drug discovery for this rare tumor has attracted significant interest from investigators with approaches such as drug repositioning, computational-assisted high-throughput drug screening, and PDO-based drug screening.

### Multidimensional drug discovery strategies for chordoma

Chordoma is a type of tumor with a low-frequency mutation rate, and the number of suitable medications identified by sequencing is limited. Preclinical studies have identified several targeted medications for the systemic management of chordoma, and indicated clinical trials have provided the efficacy and safety data (101). However, no medications have been approved so far as first-line treatments for chordoma. The main obstacles for the identification of novel therapeutic avenues include the extremely low incidence of morbidity and the lack of appropriate models for preclinical research. Conventional drug discovery pipeline is long-lasting and is not cost-effective for rare diseases such as chordoma, for which new strategies are required (102).

Given the fact that chordoma has not been sufficiently covered by target-based approaches and that it is difficult for single-agent therapies to provide lasting effects, the combined use of phenotypic screening drugs may help in the development of novel therapeutic strategies. Anderson et al. examined the combination of synergistic drugs in chordoma with a machine learning method and demonstrated a synergistic effect of palbociclib and AZD2014 with afatinib for chordoma cells *in vitro* (103). Scheipl et al. (104) performed drug screening of 133 clinically approved anticancer drugs as single agents and in combination with EGFR inhibitors (EGFRis; e.g., afatinib and erlotinib) (100) and demonstrated that the combination of crizotinib, panobinostat, and doxorubicin with EGFRis showed a promising prospect of application. The histone deacetylase (HDAC) inhibitor panobinostat exerted a moderate synergistic effect when used in combination with afatinib. Although the study has not shown groundbreaking success of these treatments as monotherapeutics in solid tumors, including chordoma (104), there appears to be a role for this class in combination therapy and multi-target inhibition (104, 105).

The combined administration of different drugs has shown advantages of prolonged efficacy, avoidance or delayed occurrence of drug resistance, and synergistic effects with increased clinical outcomes. Combination screening as a translational approach will pave the way for improved personalized drug therapies for orphan diseases like chordoma.

Drug repositioning/repurposing has become an important approach to identifying new uses for already-approved regimens beyond original indications (106, 107). Previously, drug repositioning/repurposing has often been initiated by serendipitous observation of unexpected effects (108, 109). Now, however, it has gradually shifted to a rational, computer-assisted method. Computational techniques have facilitated the drug repositioning process, which is considered time-saving and cost-effective. It offers an opportunity for secondary analysis of vetted therapies with optimized pharmacokinetics and acceptable toxicity profiles for candidates with rare diseases in clinical trials (110). A deep learning strategy featured with combination and comprehensive analyses of gene expression features, signaling pathways, clinical prognoses, and pharmacochemical characteristics has promoted the implementation of precision medicine and our understanding and management of rare diseases including chordoma (102). Integrative mining approaches include ksRepo processing, Swanson’s ABC approach, Chemotext, and ROBOKOP (Reasoning Over Biomedical Objects linked Knowledge-Oriented Pathways) (111–114). Traylor et al. (115) compared the differentially expressed genes (DEGs) of chordoma tissue samples with pharmacogenomic interactions in the Comparative Toxicogenomics Database using a drug repositioning platform named ksRepo. Alves et al. (102) reported a case of knowledge-based approach for drug repurposing with chordoma by building a connection between metformin and chordoma using the ROBOKOP platform. Their results suggested a potential treatment value by targeting osteoblast differentiation.

### Personalized drug therapies for chordoma

As a typically fatal and extremely rare disease with complex molecular mechanisms, chordoma is suitable for personalized drug therapies. Individualized identification and testing of suitable agents can provide optimal application in indicated patients. Sequencing-based computational-assisted identification of molecular targeted therapies are generally accepted by patients and clinicians (8). Medications such as TKIs, CDK4/6 inhibitors, PARP inhibitors, and EZH2 inhibitors have been widely used for patients with corresponding molecular characteristics (116). However, the response of patients to medications as identified by sequencing may not be as expected, and considering the adverse effects of medications, personalized *in vivo* or *in vitro* screening of the indicated drugs with PDC, PDX, or PDO models is considered extremely valuable.

There are several obstacles that may hinder the process of personalized drug screening with PDC and PDX models. Firstly, the establishment and the passages of PDC or PDX models are time-consuming and have low success rates. Patients need to wait for about 1–3 months after subjecting the tumor tissue to drug screening, during which the disease may have already progressed (32, 36). Secondly, PDC models are highly dependent on sampling and often fail to recapitulate the heterogeneity of the tumor microstructures and microenvironments, resulting in changes to the drug response (117). The PDX model could provide more simultaneous features as parental tumors, but is more expensive and is not suitable for high-throughput screening (37, 39).

As aforementioned, Shihabi et al. (32) reported their experience in a proof-of-concept study of personalized drug discovery with PDO models. They successfully established a high-throughput drug screening platform of personalized organoids with the following advantages compared with the previous platforms: the PDOs could be established from biopsies or tumor resections and restored most features of primary tumors, which is superior to PDCs and similar to PDXs, but is more cost-effective; moreover, it is more time-saving, as the results can be available within a week from surgery (118, 119). Based on such a high-throughput platform, the authors also established a comprehensive analysis algorithm. A dot map was then generated and subjected to clinical consideration based on the combination analysis of BioAssay from the PubChem database and the WikiPathways database.

Shihabi’s research is a typical example that illustrates the considerable value of new techniques in facilitating our understanding and management of chordoma. Successful establishment of such a screening platform profited from the advantages of the models, sequences, and bioinformatics analysis techniques, finally promoting personalized precision clinical management. Research tools and modern NGS techniques served as the two “wheels” of the “chordoma motorbike.” Comprehensive use of these two “wheels” will strikingly accelerate our understanding and management of chordoma. Efforts are still needed in the future to benefit chordoma patients by building more precise disease models, determining the intrinsic mechanisms in tumor initiation and progression, and developing optimal cutting-edge technologies for clinical application.

## Author contributions

CZ, JX, and CY conceived the study. CZ, TT, TW and XY reviewed the cell line and animal model part. CZ, EZ, HG, TL and JZ reviewed the sequencing study part. CZ, ZW, QJ and HW reviewed the personalized treatment part. CZ, TT, and EZ drafted the manuscript. JX and CY reviewed and modified the Manuscript. All authors contributed to the article and approved the submitted version.

## Funding

National Natural Science Foundation of China (Award number(s): 81902732) to CZ; Shanghai Municipal Health and Family Planning Commission (Award number(s): 201840209) and Beijing Xisike Clinical Oncology Research Foundation (Award number(s): Y-HR2020MS-0751) to CY.

## Conflict of interest

The authors declare that the research was conducted in the absence of any commercial or financial relationships that could be construed as a potential conflict of interest.

## Publisher’s note

All claims expressed in this article are solely those of the authors and do not necessarily represent those of their affiliated organizations, or those of the publisher, the editors and the reviewers. Any product that may be evaluated in this article, or claim that may be made by its manufacturer, is not guaranteed or endorsed by the publisher.
